# Micronutrient intake from three popular diet patterns in the United States: modeled replacement of foods highest in added sugar and sodium using the National Health and Nutrition Examination Survey, 2005–2018

**DOI:** 10.3389/fnut.2023.1217774

**Published:** 2023-10-13

**Authors:** Avonti Basak Tukun, Sarah Rowe, LuAnn K. Johnson, David C. Love, Martha Belury, Zach Conrad

**Affiliations:** ^1^OSU Nutrition Interdisciplinary Graduate Program, The Ohio State University, Columbus, OH, United States; ^2^College of Arts and Sciences, William & Mary, Williamsburg, VA, United States; ^3^Independent Contractor, Warren, MN, United States; ^4^Johns Hopkins Center for a Livable Future, Johns Hopkins University, Baltimore, MD, United States; ^5^Department of Environmental Health and Engineering, Johns Hopkins Bloomberg School of Public Health, Baltimore, MD, United States; ^6^Department of Food Science and Technology, The Ohio State University, Columbus, OH, United States; ^7^Department of Kinesiology, William & Mary, Williamsburg, VA, United States; ^8^Global Research Institute, William & Mary, Williamsburg, VA, United States

**Keywords:** NHANES, popular diet, micronutrient intake, low grain diet, restricted-carbohydrate diet, time-restricted diet

## Abstract

**Introduction:**

Fifty-two percent of adults in the United States reported following a popular diet pattern in 2022, yet there is limited information on daily micronutrient intakes associated with these diet patterns. The objective of the present study was to model the impact on micronutrient intake when foods highest in added sugar and sodium were replaced with healthier alternatives to align with the Dietary Guidelines for Americans recommendations.

**Methods:**

Dietary data were acquired from 34,411 adults ≥ 20 y in the National Health and Nutrition Examination Survey, 2005–2018. The National Cancer Institute methodology was used to estimate usual dietary intake at baseline of 17 micronutrients using information from up to two dietary recalls per person. A food substitution model was used to evaluate the impact on micronutrient intake when three servings of foods highest in added sugar and sodium were substituted with healthier alternatives.

**Results:**

Dietary modeling to replace foods highest in added sugar with healthier alternatives increased the mean intake of fat-soluble vitamins (0.15% for vitamin A to 4.28% for vitamin K), most water-soluble vitamins (0.01% for vitamin B_1_ to 12.09% for vitamin C), and most minerals (0.01% for sodium to 4.44% for potassium) across all diet patterns. Replacing foods highest in sodium had mixed effects on the mean intake of micronutrients. The intake of most fatsoluble vitamins increased by 1.37–6.53% (particularly vitamin A and D), yet while the intake of some water-soluble vitamins and minerals increased by 0.18–2.64% (particularly vitamin B_2_, calcium, and iron) others decreased by 0.56–10.38% (notably vitamin B_3_ and B_6_, magnesium, sodium, and potassium).

**Discussion:**

Modeled replacement of foods highest in added sugar led to more favorable changes in mean micronutrient intake compared to modeled replacement of foods highest in sodium. Due to the composite nature of mixed dishes that include multiple ingredients, food substitutions may result in both favorable and unfavorable changes in micronutrient intake. These findings highlight the challenges of making singleitem food substitutions to increase micronutrient intake and call for further research to evaluate optimal combinations of replacement foods to maximize the intake of all micronutrients simultaneously.

## 1. Introduction

The proportion of people following popular diet patterns, which typically modify the intake of macronutrients (e.g., restricted-carbohydrate), food groups (e.g., vegetarian), or eating time (e.g., time-restricted), in the United States (US) has risen from 38% in 2019 to 52% in 2022 (1, 2). During the 2005–2018 period, an average of 44% of people in the US tried at least one popular diet pattern on a given day that restricted macronutrients, food groups, or eating time (3).

Prior research has demonstrated that the overall quality of popular diet patterns when measured using the Healthy Eating Index (HEI)-2015–which measures adherence to the Dietary Guidelines for Americans (DGA)–is poor, ranging from 52–65 points out of 100 (3, 4). When these diets were modified by replacing foods highest in added sugar, sodium, saturated fat, and refined grains with healthier alternatives, diet quality improved modestly (2.5 to 9.8 points), pulling HEI scores slightly above the average score of the US population (58 out of 100), but all diet patterns remained far below optimal (3). Others have shown that dietary modeling to accommodate DGA recommendation had heterogenous effect on the intake of food groups, such as elevating the intake of fruits and vegetables, respectively, up to 47% and 10%, but decreasing the intake of dairy by as much as 11%, across different popular diet patterns (5). To our knowledge, dietary modeling has not been used to assess micronutrient intake in popular diet patterns that restrict food groups, macronutrients, or eating time.

The 2020–2025 DGA recommends limiting the intake of foods high in added sugar and sodium and increasing the intake of fruits, vegetables, whole grains, and sources of unsaturated fats (6). About 13% of the total energy intake of US adults is derived from added sugar (about 17 teaspoons or 85 grams per day) whereas the DGA-recommended intake is <10% (6–8). Most adults (about 90%) have very high sodium intake with a mean of 3,468 mg/day, 51% higher than the Chronic Disease Risk Reduction (CDRR) (2,300 mg/day) (9). People in the US who consume more than 10% of energy from added sugar have a 7–103% increased risk of death from cardiovascular diseases (CVD) compared to those who consume 7.4% energy from added sugar (10). Others have shown that CVD risk increases by 17% for every additional 1,000 mg/day of sodium intake (11).

Many foods are consumed as mixed dishes that contain multiple ingredients, so limiting the intake of foods high in added sugar and sodium may also impact the intake of some micronutrients. Hence, the purpose of any food substitution to comply with the DGA recommendation is not only to improve a single component in diet but also to improve overall diet quality, including micronutrient intake. It is therefore critical to evaluate whether replacing foods highest in added sugar and sodium with healthier alternatives impacts micronutrient intake among those that follow popular diet patterns. To address this research need, the present study models the changes in the mean daily intake of 17 micronutrients for three popular diet patterns (low grain, restricted-carbohydrate, and time-restricted) in the US when 3 servings of foods highest in added sugar or sodium were replaced with healthier alternatives as per the DGA recommendations.

## 2. Materials and methods

### 2.1. Data acquisition

Individual-level data on food intake, nutrient intake from foods and supplements, and sociodemographic status were retrieved from the National Health and Nutrition Examination Survey (NHANES), 2005–2018. NHANES collects data from approximately 5,000 non-institutionalized participants per year using a multi-stage, stratified, clustered sampling design, and some population groups are oversampled (12). Trained interviewers collect dietary data using the computer-assisted Automated Multiple Pass Method to minimize respondent burden and increase reliability and validity (13, 14). Most (80%) participants complete a second recall by telephone 3–10 days later (15). The salt adjustment was appropriately removed from dietary data collected from 2005–2008 to standardize measurement of sodium intake across all data years (16). Many participants reported consuming mixed dishes that include multiple food groups, so the Food Patterns Equivalents Database (FPED) was used to convert each NHANES mixed dish into one or more food groups that represent those included in the DGA (17). This study is a secondary analysis of de-identified and publicly available data and was exempted from human studies ethical review by the Institutional Review Board at William & Mary. Pre-registration for this study can be found elsewhere (18).

### 2.2. Baseline (current) dietary intake

The National Cancer Institute’s (NCI) usual intake methodology was used to estimate current (baseline) dietary intake of kcal and nutrients. This method estimates within-person variation of the entire sample using data from two 24-h recalls collected from most participants (19). The SAS macros MIXTRAN (v2.21) and INDIVINT (v2.3) were used to estimate predicted intake at the individual level. The probability of consumption was assumed to be correlated to the amount consumed for nutrients consumed episodically (20); which include the polyunsatured fatty acids, namely eicosapentaenoic acid (20:5) and docosahexaenoic acid (22:6). The SAS macros NLMIXED_UNIVARIATE (v1.2), NLMIXED_BIVARIATE (v1.2) and PREDICT_INTAKE_DENSITY (v1.2) were used to estimate nutrient densities (e.g., percent energy from carbohydrate) (21–23).

### 2.3. Diet pattern categorization

Each participant was categorized into one or more diet patterns using data on usual food and nutrient intake at baseline: low grain, restricted-carbohydrate, and time-restricted. Supplementary Table 1 describes the characteristics of these diet patterns, which was informed by published literature (24–28). Low grain, restricted-carbohydrate, and time-restricted diet patterns were categorized, respectively, using data on daily intake of food groups from FPED, daily nutrient intake from NHANES nutrient files, and the amount of time between each eating occasion for each participant from the NHANES individual foods file. It was possible for participants to be categorized into more than one diet pattern. The NCI methodology does not predict zero intake, so non-consumers were identified if they did not consume any amount of a given food group on both days of recall. To provide relevance to the general population, an additional diet pattern was established that included all participants that met the inclusion criteria specified below, regardless of whether they were categorized into one of the dietary patterns described above.

### 2.4. Food categories and serving sizes

The categorization scheme used by the Food and Nutrient Database for Dietary Studies (FNDDS) (29) and FPED (17) was used to categorize each food and beverage consumed by participants on the first day of dietary recall into 89 mutually exclusive categories. For example, pasta dishes were identified using FNDDS and were further disaggregated into whole grain and refined grain pasta dishes using FPED (Supplementary Table 2). Data on the gram weight of each food consumed as well as their kcal and nutrient content were acquired from NHANES files, and data on the amount of added sugar present in each food were acquired from FPED files. Average serving sizes of each food category were estimated from these data, as well as the average amount of kcal, added sugar, and sodium present per serving of each food category, as described below.

Serving sizes for each food category were estimated for the entire sample by averaging the gram weight of each food consumed within each category at each eating occasion. For example, there were 174 types of refined grain pasta dishes that were consumed on 5,424 occasions, and the average amount (in grams) of refined grain pasta dishes consumed at each eating occasion was used as the serving size for this food category. All computed serving sizes for packaged foods and beverages were consistent with serving sizes on the Nutrition Facts Panel of these products (30), and the computed serving sizes for non-packaged foods were consistent with serving sizes provided by FPED documentation (31). To estimate the amount of kcal, nutrients, and added sugar per serving of each food category, the amount of these food components per gram of each food was averaged across all foods within each food category and was multiplied by the average serving size (in grams) of each food category.

### 2.5. Target foods and alternative foods

For each diet pattern, food categories to be removed during modeling (target foods) were those that represented the greatest daily intake of added sugar and sodium using data from the first day of dietary recall. Alternative food categories were selected based on four criteria: (1) adhered to the principles of each diet pattern (e.g., a grain dish was not used as an alternative food for the low grain diet pattern), (2) represented a reasonable dietary substitution that an individual may make, as determined through consultation and consensus with multiple Registered Dietitian Nutritionists (RDN) as described by Conrad et al. (3) (e.g., dishes were replaced with dishes, beverages with beverages, snacks with snacks, and desserts with desserts), (3) it was consumed in the greatest quantity of the remaining options, and (4) it had a lower content of added sugar and sodium per serving compared to the target food.

### 2.6. Diet modeling

A diet model was constructed to evaluate the effects on nutrient intake if up to 3 servings of food categories highest in added sugar or sodium (target foods) were substituted with up to 3 servings of alternative foods, which is consistent with DGA 2020–2025 recommendations to limit these components in the diet (32). To allow for discretionary intake, the model only performed substitutions if a participant consumed at least the number of servings of the target food compared to the alternative food. For example, if a participant consumed 0.9 servings of a target food it would not get substituted with an alternative food, but if they consumed 1.1 servings of a target food it would get substituted with 1 serving of an alternative food. Similarly, if a participant consumed 2.9 servings of a target food it would get substituted with 2 servings of an alternative food. All substitutions were made on the basis of whole servings (1, 2, or 3 servings) rather than mass quantity or kcal to reflect the units in which individuals typically consume foods and beverages. Individual-level intake of kcal and nutrients were estimated at baseline and after modeling.

### 2.7. Statistical analyses

Micronutrient intake was evaluated at baseline (i.e., current intake) and after modeling. Means were unadjusted and tests of significance were adjusted for age, sex, and NHANES survey cycle using linear regression models. Survey weights and design variables from NHANES were used to account for the multistage probability sampling design and to produce nationally representative estimates. SAS 9.4 (SAS Institute; Cary, NC, USA) was used to estimate usual intakes using the NCI macros, and Stata 16.1 (StataCorp; College Station, TX, USA) was used for data management and all other analyses.

## 3. Results

### 3.1. Sample characteristics

Participants were excluded from the present study if they were less than 20 years old (*n* = 26,375) or pregnant or lactating women or did not provide complete and reliable dietary information (*n* = 896). Of the 61,682 participants in NHANES 2005–2018 that provided dietary data, 34,411 participants met the inclusion criteria.

Details of the participant characteristics are included in Table 1. In the general population group, the mean age of participants was 48 years, approximately half were female (51%), and the majority were non-Hispanic white (67%). The mean income-to-poverty ratio of those in the general population was 3.0, and 61% of the participants attended some college. Of participants that were categorized into a popular diet pattern, most followed a restricted-carbohydrate diet (29%), followed by low grain (10%), and time-restricted (9%) (Table 1). Participants that followed a low grain diet pattern had a mean age of 50 years, were mostly female (70%) and non-Hispanic white (70%), 59% attended some college, and had a mean income-to-poverty ratio of 2.8. Participants that consumed a restricted-carbohydrate diet pattern had a mean age of 48 years, over half were male (56%), most were non-Hispanic white (73%) and attended some college (66%), and had a mean income-to-poverty ratio of 3.3. Participants that followed a time-restricted diet were younger (43 years), had less educational attainment (48% attended college), and had a lower income-to-poverty ratio (2.4). About half of the participants in the time-restricted diet pattern group were female (52%) and non-Hispanic white (52%).

**TABLE 1:** Characteristics of study participants, 2005–2018 (*n* = 34,411).

Characteristic	General population^1^ (*n* = 34,411)	Low grain^2^ (*n* = 3,446)	Restricted-carbohydrate^3^ (*n* = 9,025)	Time-restricted^4^ (*n* = 4,115)
**Mean or % (95% CI)^5^**				
Percent of population, %	100.0	10.2 (9.7–10.7)	28.7 (27.8–29.6)	9.2 (8.7–9.7)
Age, y, %	47.8 (47.3–48.3)	50.1 (49.4–50.9)	47.8 (47.2–48.3)	42.6 (41.7–43.5)
Female, %	51.0 (50.3–51.6)	69.5 (67.3–71.7)	44.0 (42.6–45.4)	51.9 (49.6–54.2)
At least some college, %	60.5 (58.8–62.2)	59.2 (56.4–62.0)	65.7 (63.7–67.7)	48.1 (45.2–50.9)
Income-to-poverty ratio, %	3.0 (2.9–3.1)	2.8 (2.7–2.9)	3.3 (3.2–3.4)	2.4 (2.3–2.5)
Race/ethnicity, %				
Non-Hispanic white, %	67.3 (64.7–69.7)	69.5 (66.1–72.6)	73.4 (71.0–75.7)	52.5 (48.7–56.4)
Non-Hispanic black, %	11.3 (10.0–12.8)	14.9 (12.9–17.3)	10.7 (9.4–12.1)	21.8 (19.1–24.9)
Other, %	21.4 (19.6–23.3)	15.6 (13.7–17.7)	16.0 (14.3–17.7)	25.6 (22.9–28.5)

Adapted with permission from Conrad et al. (3). Quality of popular diet patterns in the United States: evaluating the effect of substitutions for foods high in added sugar, sodium, saturated fat, and refined grains. Current developments in nutrition, 12:nzac119.

Sample sizes are unweighted.

^1^All participants that met inclusion criteria, including those in each diet category as well as those not categorized into diet categories.

^2^≤ 10th percentile of total grain intake.

^3^< 45% kcal from carbohydrate.

^4^≥ 12 h food and beverage fast.

^5^Age and income-to-poverty ratio values are means, values for all other characteristics are percents. All values are adjusted for survey weights and multistage survey design.

### 3.2. Sources of added sugar and sodium

All diet patterns evaluated in the present study had the same food sources that were highest contributors of added sugar (soft drinks with added sugar) and sodium (poultry dishes; Supplementary Table 3). Soft drinks with added sugar contributed to 17.9–35.6% of daily intake of added sugar for all diet patterns and poultry dishes contributed to 8.5–9.5% of daily sodium intake for all diet patterns. To generate modeled diets, soft drinks with added sugar were replaced by soft drinks without added sugar in the general population, restricted-carbohydrate, and time-restricted diet patterns, but were replaced by 100% fruit juice in the low grain diet pattern. In the case of modeled diets for sodium, poultry dishes were substituted with egg dishes in all diet patterns. The nutrient content per serving of target food items and their healthier alternatives are presented in the Supplementary Table 4.

### 3.3. Micronutrient intake at baseline

Mean baseline intake of nutrients and energy are presented in Supplementary Tables 5–8. Intakes of all fat-soluble vitamins were lower among participants that followed a time-restricted diet pattern than the general population. The low grain diet pattern had lower mean intake of vitamin A (825.51 μg; 95% CI: 786.98, 864.05) and vitamin E (7.11 IU; 6.94, 7.3) compared to the general population, whereas the restricted-carbohydrate diet pattern had higher mean intake of vitamin E (8.64 IU; 8.53, 8.75) and vitamin K (114.89 μg; 113.34, 116.44) than the general population. Similar to the fat-soluble vitamins, intakes of all water-soluble vitamins were lower among participants that followed a time-restricted diet pattern compared to the general population. The mean intakes of folate among participants in each of the three popular diet patterns (454.74–575.92 μg) were lower compared with the general population (614.80 μg; 609.38, 620.22). Compared to the general population, participants that followed a low grain diet pattern had a lower mean intake of vitamin B_3_ (25.43 mg; 24.43, 26.43), and participants that followed a restricted-carbohydrate diet pattern had a lower intake of vitamin B_6_ (4.39 mg; 4.07, 4.71) but a higher intake of vitamin B_3_ (34.18 mg; 33.3, 35.05) compared to the general population.

The intake of six minerals (calcium, magnesium, iron, zinc, sodium, and potassium) were lower in the low grain and time-restricted diet patterns compared to the general population. The restricted-carbohydrate diet pattern had higher mean intake of magnesium (337.15 mg; 332.64, 341.66), zinc (15.29 mg; 15.07, 15.5), sodium (3,705.48 mg; 3,677.95, 3,733.01), and potassium (2,735.06 mg; 2,711.45, 2,758.67) than the general population.

### 3.4. Micronutrient intake in response to modeled replacement of foods highest in added sugar

**FIGURE 1:**
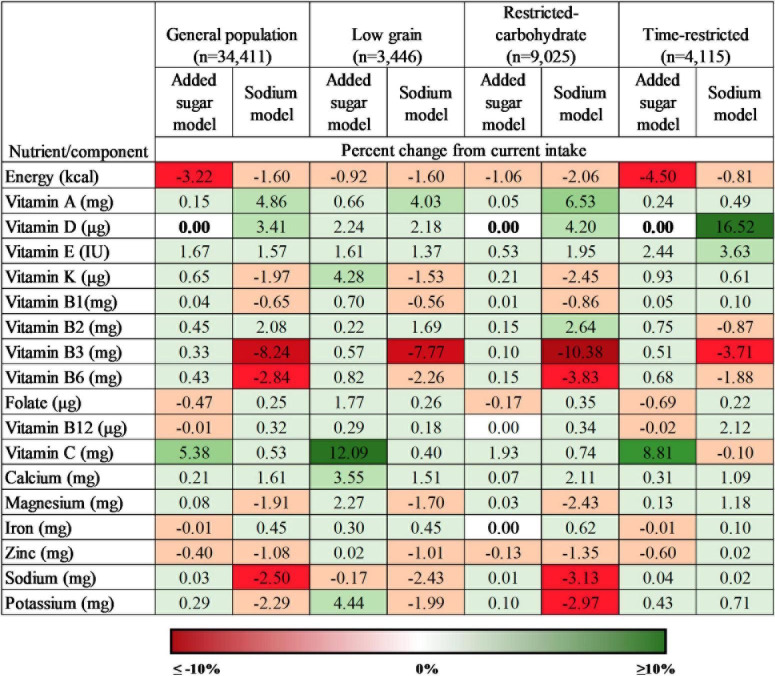
Percent change in intake of energy and micronutrients under conditions of modeled food replacement for popular diet patterns and the general population, 2005–2018 (*n* = 34,411). All values are statistically significant at *P* < 0.05 using paired Wald tests, except those in bold text.

Figure 1 presents the percent changes in mean micronutrient intake after modeling, and Supplementary Tables 5–8 presents the mean and the 95% confidence interval (CI) of micronutrient intakes before and after modeling. Modeled replacement of foods highest in added sugar with healthier alternatives led to reduced energy intake (0.92–4.5%) and increased intake of most micronutrients across popular diet patterns (0.01–12.09% increase) and the general population (0.03–5.38% increase). The highest increase among fat-soluble vitamins was estimated for vitamin K (4.28%; corresponding to 4.55 μg increase from baseline) in the low grain diet pattern, which also had the highest increase for vitamin C intake (12.09%; 20 mg). The lowest change in vitamin C intake was estimated for the restricted-carbohydrate diet pattern (1.93% increase), corresponding to a 2.97 mg increase from baseline (154 mg). Of the minerals, the highest change in intake was estimated for potassium (4.44%; 104.86 mg increase), followed by calcium (3.55%; 31.08 mg increase) among participants with a low grain diet pattern. The rest of the changes in micronutrient intakes were relatively small (0.01–2.44%), especially when there were decreases in intake (less than 1%). No changes were evident for vitamin D intakes among participants in the restricted-carbohydrate and time-restricted diet patterns, and among participants in the general population; and no changes were observed for vitamin B_12_ and iron intakes among participants in the restricted-carbohydrate diet pattern. All changes in the energy intake and intakes of micronutrient are statistically significant (*p* < 0.05 using paired Wald tests), except for the vitamin D.

### 3.5. Micronutrient intake in response to modeled replacement of foods highest in sodium

Modeled replacement of sodium rich foods (Figure 1 and Supplementary Tables 5–8) decreased mean energy intake (1.6–2.06%) as well as increased mean intake of vitamins A (4.03–6.53%; corresponding to 33–58 μg), D (2.18–5.77%; 0.43–0.54 μg), and E (1.37–1.95%; 0.1–0.17 IU), and decreased intakes of vitamin K (1.53–2.08%; 1.63–1.95 μg), vitamin B_1_ (0.56–0.86%; 0.02–0.03 mg), vitamin B_3_ (7.77–10.38%; 1.98–3.55 mg), B_6_ (2.26–3.83%; 0.1–0.17 mg), magnesium (1.7–2.43%; 4.73–8.19 mg), zinc (1.01–1.21%; 0.12–0.14 mg), sodium (2.43–3.13%; 67–116 mg), and potassium (1.99–2.5%; 47–56 mg) among all diet patterns. The maximum increase in water-soluble vitamins was estimated for vitamin B_2_ (2.64%; 0.1 mg) for the restricted-carbohydrate diet pattern. Among the six minerals assessed in this study, only calcium (1.51–2.11%; 13.21–22.85 mg) and iron (0.45–0.62%; 0.06–0.1 mg) increased across all diet patterns. All results were statistically significant (*p* < 0.05) using paired Wald tests.

## 4. Discussion

In this nationally representative modeling study of individuals that consumed popular diet patterns, replacing foods highest in added sugar and sodium with healthier alternatives reduced energy intake and increased intake of many micronutrients, with some exceptions. Modeled replacement of foods highest in added sugar led to a modest decrease in the mean intake of folate, vitamin B_12_, iron, and zinc for most diet patterns, and modeled replacement of foods highest in sodium led to larger decreases in the mean intake of vitamins B_3_ and B_6_ for most diet patterns. These findings highlight the challenges of making single-item food substitutions to increase micronutrient intake, which can lead to favorable changes in intake of some micronutrients but not others.

In the US, high consumption of added sugar and sodium is associated with elevated risk of morbidity and mortality from obesity and CVD (33). Added sugar consumption in the US decreased from 18% of total energy in 1999–2000 (34) to 13% in 2017–2018 (35), but remained above the recommended threshold of 10% (6). In the present study, added sugar contributed to more than 10% of total energy, except for participants that consumed the restricted-carbohydrate diet pattern. Soft drinks are the principal source of added sugar in the diet of adults in the US (7, 36), which was also confirmed in the present study. After modeled replacement, the percentage of total energy from added sugar decreased by 0.7 to 2.8 percentage points across all diet patterns. Almost 90% of US adults exceed the CDRR threshold for daily sodium intake (9, 37) of 2,300 mg/day. Dietary modeling to substitute foods highest in added sugar and sodium with healthier alternatives reduced energy intake as expected, but dietary modeling to substitute three servings of sodium-rich items was not enough to bring sodium intake below 2,300 mg/day. To have a significant reduction in CVD related mortality in the US, reducing daily sodium intake by 40% or to 1,500 mg has been proposed (38), whereas the present study estimated less than a 5% (corresponds to 5–115 mg) reduction.

Factors that drive the extent of changes in micronutrient intake in the modeled diet patterns are the nutrient composition of replaced and alternative foods, the content of undesired food components in alternative foods, and the mean intake of micronutrients at baseline. The nutrient composition of the replaced items and the alternative items determines the level of change in micronutrient content of modeled diets. For instance, others showed that replacing typical breakfast foods with ready-to-eat cereals improved the intake of several micronutrients (vitamin D, folate, iron, and dietary fiber), and an additional increase for calcium and potassium was achieved when milk was added (39).

Modeled diet patterns that replaced foods highest in added sugar with soft drinks without added sugar led to moderately lower intakes of folate (measured as dietary folate equivalent; 0.17–0.69%), vitamin B_12_ (0.01–0.02%), iron (0.01%), and zinc (0.04–0.06%). However, in the low grain diet pattern, foods highest in added sugar (soft drinks with added sugar) were replaced with 100% fruit juice which resulted in increased intake of all micronutrients (0.02–12.09%, with the highest increase observed for vitamin C). The high vitamin C content of 100% fruit juices (40) (Supplementary Table 4) contributed to the increase of vitamin C in the modeled diet patterns, which might also be true for the increase in other micronutrients as micronutrient content per serving of the 100% fruit juice is higher than that of soft-drinks without added sugar. Similarly, the nutrient compositions of soft drinks with added sugar and soft drinks without added sugar are similar, except for energy and a few minerals (calcium, magnesium, and potassium) (Supplementary Table 4), which may have led to minute or no changes in intake of vitamins (e.g., vitamin D, K, B_1_, B_2_, B_12_) and minerals (iron, zinc, calcium, sodium, etc.) after dietary modeling in the general population, restricted-carbohydrate, and time-restricted diet patterns.

Improving the intake of a single dietary component might increase the intake of other undesired food components (41) and limit the intake of under-consumed micronutrients. In the present study, modeled replacement of foods highest in sodium (poultry dishes) with healthier alternatives (egg dishes) increased the intake of saturated fat (1.43–1.75%) and cholesterol (12.6–17.37%). Similarly, modeled replacement of foods highest in sodium increased the intake of several under-consumed micronutrients (e.g., vitamin A, vitamin D, folate, and calcium), but at the same time it decreased the intake of several important micronutrients such vitamin B_3_, vitamin B_6_, and potassium. The mixed nature of replacement dishes (i.e., including nutrients to encourage as well as nutrients to limit) may result in both favorable and unfavorable changes. This is consistent with a similar study that assessed changes in consumption of food groups using modeled substitutions, and found an increase in modeled intake of fruits and whole grains but a decrease in modeled intake of vegetables in some popular diet patterns (5). Further research is needed to utilize optimization modeling to identify food substitutions that lead to favorable changes in all or most micronutrients.

Finally, because the present study used the percentage change from baseline intake as the unit of measure, the level of intake of a micronutrient at baseline affected the percent increase in the modeled diets. For example, identical food items with equivalent weight were substituted, yet a lower baseline intake of vitamin C among participants that consumed time-restricted diet patterns (122.05 mg) than participants that consumed restricted-carbohydrate diet patterns (154.0 mg) resulted in a higher increase in vitamin C intake (8.8 vs. 1.93%) after dietary modeling.

This study provides a foundation for future studies that could evaluate intake of micronutrient from popular diet patterns. Other tools are available to examine micronutrient intake, including indices that measure the intake of multiple nutrients collectively, such as total nutrient intake (TNI), mean probability of adequacy (MPA), and the Nutrient Rich Food Index 9.3 (NRF9.3) (42, 43). Further research could apply these tools to popular diet patterns and could be used to gauge the extent of achievable improvement through modeled replacement with different foods. In addition, studies could estimate the proportion of people within each diet pattern at risk of developing micronutrient inadequacy by comparing with the Recommended Dietary Allowance (RDA) and the Tolerable Upper Intake Level (UL), which can help inform dietary interventions. It will also be useful to compare different alternative foods, especially those with and without artificial sweeteners as the World Health Organization (WHO) suggests that long-term intake of high amounts of artificial sweeteners may present adverse health effects (44).

The present study has several strengths. To the authors’ knowledge, this is the first study to report the mean micronutrient intake of low grain, restricted-carbohydrate, and time-restricted diet patterns in the US. Data were collected from a large, nationally representative sample of over 34,000 participants over a 14-year period, making these findings generalizable to the US population. For the first time, this study also evaluated the changes in micronutrient intake for people following popular diet patterns when foods highest in added sugar and sodium were replaced with healthier alternatives. Diet patterns were categorized using data from multiple 24-h recalls instead of participants’ self-reported adherence to diet patterns, because mischaracterization of diet patterns by study participants is common (45, 46). Serving sizes were also estimated from this nationally representative data and were consistent with those provided on the Nutrition Facts Panel (30) and published in the United States Department of Agriculture (USDA) FPED (17). Additionally, the use of a serving in lieu of mass quantity reflects the units in which most people make food substitutions.

This study also has some limitations. As with all self-reported dietary data, 24-h recalls are subject to recall bias and social desirability bias which might lead to under- or over-estimation of micronutrient intake (47, 48). However, 24-h dietary recalls provide valuable and highly detailed dietary information and are especially useful when assessing dietary information from large samples (47, 48). This study evaluated replacing one target food with one alternative food, and other substitutions may yield different results. Similarly, substitution with alternative foods of equal weight or energy could produce different results than the present study. The present study included three popular diet patterns, and other diet patterns such as vegetarian and pescatarian were not included since less than 3% of the participants in our sample followed them, which has been reported elsewhere (3). However, diet patterns that are commonly reported but have low adherence in practice could be a potent future research area to explore. Also, micronutrient intake was not assessed by any nutrient adequacy index in the present study, thus future research could use such nutrient indices to evaluate micronutrient adequacy of popular diet patterns for guiding people to improve nutrition and health outcomes. Finally, these results represent average dietary changes of population subgroups which can produce overgeneralized results for some individuals.

## 5. Conclusion

Dietary modeling to replace foods highest in added sugar and sodium with healthier alternatives, as per DGA 2020–2025 recommendations, had heterogeneous effects on mean micronutrient intake across popular diet patterns. Modeled replacement of foods highest in added sugar led to more dramatic improvements in micronutrient intake with more modest reduction of energy intake than replacement of foods highest in sodium. When interpreting these results, one should consider the difference in nutrient composition of replacement foods, which determines the extent of benefits for micronutrient intake. The results of the present study give directions for future research regarding micronutrient adequacy of popular diet patterns. Optimization modeling approaches may be useful in estimating the appropriate combinations of different alternative foods items to maximize micronutrient adequacy.

## Data share policy

Data described in this manuscript, code book, and analytic code will be made publicly and freely available without restriction at https://archive.org/details/osf-registrations-z3fme-v1. This study was pre-registered at the Center for Open Science, Open Science Framework at https://archive.org/details/osf-registrations-z3fme-v1.

## Data availability statement

Publicly available datasets were analyzed in this study. This data can be found here: https://wwwn.cdc.gov/nchs/nhanes/.

## Ethics statement

Ethical review and approval were waived for this study because it is a secondary analysis of publicly available and de-identified data (William & Mary Institutional Review Board, PHSC-2022-03-02-15499-zsconrad, 03/02/2022).

## Author contributions

ZC and MB: conceptualization, methodology, and funding acquisition. ZC and LJ: software, validation, formal analysis, and investigation. ZC: resources, supervision, and project administration. ZC, ABT, SR, and LJ: data curation. ABT: writing—original draft preparation. ZC, ABT, and DL: visualization. All authors wrote, reviewed and edited the manuscript, read, and agreed to the published version of the manuscript.
